# Inequality in Healthcare Utilization in Italy: How Important Are Barriers to Access?

**DOI:** 10.3390/ijerph19031697

**Published:** 2022-02-01

**Authors:** Domenica Matranga, Laura Maniscalco

**Affiliations:** 1 Department of Health Promotion, Mother and Child Care, Internal and Medical Specialties “G. D’Alessandro”, University of Palermo, 90127 Palermo, Italy; 2 Department of Biomedicine, Neuroscience and Advanced Diagnostics, University of Palermo, 90127 Palermo, Italy; laura.maniscalco04@unipa.it

**Keywords:** healthcare, utilization, financial barriers, waiting list, access, inequality, horizontal equity

## Abstract

With the ageing population, equitable access to medical care has proven to be paramount for the effective and efficient management of all diseases. Healthcare access can be hindered by cost barriers for drugs or exams, long waiting lists or difficult access to the place where the needed healthcare service is provided. The aim of this paper is to investigate whether the probability of facing one of these barriers varies among individuals with different socio-economic status and care needs, controlling for geographical variability. Methods. The sample for this study included 9629 interviews with Italian individuals, aged 15 and over, from the second wave (2015) of the European Health Interview Survey, which was conducted in all EU Member States. To model barriers to healthcare, two-level variance components of logistic regression models with a nested structure given by the four Italian macro-areas were considered. Results. Of the barriers considered in this study, only two were found to be significantly associated with healthcare utilization. Specifically, they are long waiting lists for specialist service accessibility (adjOR = 1.20, 95% CI (1.07; 1.35)) and very expensive exams for dental visit accessibility (adjOR = 0.84, 95% CI (0.73; 0.96)). Another important result was the evidence of an increasing north–south gradient for all of the considered barriers. Conclusion. In Italy, healthcare access is generally guaranteed for all of the services, except for specialist and dental visits that face a waiting time and financial barriers. However, barriers to healthcare were differentiated by income and sex. The north–south gradient for healthcare utilization could be explained through the existing differences in organizational characteristics of the several regional healthcare services throughout Italy.

## 1. Introduction

The Italian National Health Service (INHS) promotes equity and the reduction of geographic inequalities in the supply of services. INHS ensures high quality in healthcare services related to hospital admissions, emergency care, the services of general practitioners (GPs), and pediatricians. In addition, its main source of financing is national and regional taxes, supplemented by co-payments for pharmaceuticals and outpatient care [1]. The principles are related to universal coverage and non-discriminatory access to healthcare services. INHS guarantees the health right to all people, without discrimination based on income, gender or other social status factors. The general objectives and fundamental principles for the INHS, together with the so-called “essential levels of care” (LEA Decree, dated 29 November 2001) are set at the national level. Every year, through the LEAs, the Ministry of Health selects the categories of services delivered at the hospital level to all of the citizens and allocates national funds to the regions. Regional governments are responsible for administrative healthcare functions, planning healthcare activities, organizing supply in relation to population needs, as well as monitoring the quality, appropriateness, and efficiency of the services provided. At the local level, local health authorities are directly responsible for primary care as well as secondary and specialist care or through public hospitals or accredited private providers [1].

To assess healthcare quality, a multidimensional approach should include not only appropriateness and efficiency [2], but also equitable access to care [3]. Horizontal equity consists of the requirement of equal opportunities to access healthcare for those in equal need of care. Equal opportunities require that services, when needed, are available, and the applicants do not face any financial, organizational, social or cultural barrier [4].

In general, access and utilization are used as synonyms, but they have different meanings. Access is defined as the opportunity, while usage is the proof that the subject can access a service. Using utilization to determine access could exclude important barriers, which are essential for understanding possible inequalities [4].

In Europe, less deprived people have more access to secondary care services [5,6,7,8,9,10]. In addition, physician utilization for outpatients with different SES varies more for specialist visits than for GPs [6,8,9,10]. The current COVID-19 pandemic has exacerbated socio-economic inequalities in access to care. Poor people with low levels of education as well as overcrowded and poor hygienic housing conditions have been more broadly exposed to the infection. In addition, they have experienced less access to testing, self-protection devices, and reported worse results [11].

Similar to other European countries, the ageing population and the difficult economic situation is expected to put pressure on public expenditure on health and long-term care in the near future. In Italy, different healthcare access barriers have been expressed at the geographical level, to the detriment of the central and southern regions, and at the socio-demographic level, to the detriment of the most disadvantaged social strata, such as the less educated and poor [12]. In an Italian study, inequitable access due to financial reasons was found for all of the healthcare services, except for hospitalization. Furthermore, access to GPs, specialists, and hospital admission was affected by geographical inequities and educational disparities [10]. With respect to dental care, a recent review regarding social inequalities in the number of natural teeth in the over 50-year-old European population ranked Italy at the 6th position on the concentration index by income and education [13].

To measure barriers to healthcare utilization, the European Health Interview Survey (EHIS) [14] calculates several indicators. The questions investigate whether an individual’s medical need is not met due to the cost of a drug or an exam, long waiting times or difficult access to the place where the needed healthcare service is provided (geographic availability).

This paper aims to explore whether and how the probability of encountering one of the above-mentioned barriers varies among individuals with different SES and healthcare utilization, such as a visit to the GP, to a specialist, hospitalization or a dental visit. For further analysis, the magnitude of inequity in the access to a service will be assessed, controlling for geographical repartition into North–East, North–West, Centre and South-Islands [15]. This classification reflects the north–south gradient for socio-economic and cultural differences, as well as for the quality of life, health, and healthcare [12].

## 2. Materials and Methods

### 2.1. Study Population and Variables

Data for this study were obtained from the second wave (2015) of the EHIS, which was conducted in all EU Member States. The sample included 9629 interviews with Italian people, aged 15 and over. For the scope of this analysis, data were considered as individuals nested within the geographical area of residence and analyzed through multilevel models.

The EHIS focuses on three thematic areas: Health status, health determinants, and access to and use of the health services investigated. The analyzed dataset contains demographic and socio-economic status variables: Sex (female as reference, male), age (15–24 as reference, 25–64, 65+), education (no qualification as reference, middle, high, graduation), labor status (unemployed as reference, employed, retired, other), and income, measured through the net monthly equivalized income of the household, converted into quintiles. The 1st quintile identifies the lowest income (reference), whereas the 5th quintile identifies the highest income.

The health status variables include self-perceived health (less than good as reference, good, and very good), chronic conditions (none as reference, at least one), and physical limitation in daily activities (none as reference, at least one).

Healthcare utilization is measured as the likelihood of a visit to a GP or a specialist, of hospitalization or of a dental visit in the last 12 months.

A visit to a GP was measured through the question: ‘‘When was the last time you spoke or saw a general practitioner about a health problem?”, while dental visits were measured through the question: ‘‘When was the last time you went to the dentist/orthodontist/dental hygienist for the care or hygiene of your teeth?’’. Specialist visits were elicited by the question: ‘‘When was the last time you had a specialist medical examination (ophthalmology, orthopedic, cardiological, gynecological, psychiatric, etc.) for a problem concerning your health?”, while hospitalization was assessed through the question “In the last 12 months, have you been hospitalized for at least one night?”. All of the variables were dichotomized as “yes” or “no”, respectively coded as 1 and 0. The respondents who answered “No need for healthcare” to one of the above questions were excluded from the study.

The barriers to healthcare access were measured through the following four questions, expressed as binary variables assuming value 1 for answer “yes” and 0 for “no”: (i) “In the last 12 months, did you experience delay in performing any health services (medical examinations, clinical analyses, diagnostic tests, etc.) due to a long waiting list?”; (ii) “In the last 12 months, did you experience delay in access to any health services (medical examinations, clinical analyses, diagnostic tests, etc.) due to distance or unavailability of means of transport?”; (iii) “In the last 12 months, has it ever happened that you did not perform the exams or medical treatment that you need due to difficulties of payment or high cost?”; (iv) “In the last 12 months, has it ever happened that you did not take the prescribed drugs that you need due to difficulties of payment or high cost?”

### 2.2. Statistical Methods

The continuous variables were summarized as the mean and standard deviation, median, and range. The categorical variables were analyzed as counts and percentages. To assess the association between the variables, the chi-squared test was used for categorical variables, and the ANOVA test was used for continuous variables.

Herein, considering the binary nature of the response variables to model healthcare access related to SES and services utilization and due to the stratified design used to collect the data analyzed, we used a set of two-level variance components of logistic regression models with a nested structure given by four macro-areas (North–West, North–East, Centre and South-Islands). Let $p_{ij}$ be the probability of facing one healthcare barrier for the *i*-th respondent living in macro-area *j*, thus the model is specified as follows:

$$logit\left(p_{ij}\right)=\beta_{0}+\beta_{1}X_{1\;ij}+\beta_{2}X_{2\;ij}+\ldots +\beta_{p}X_{k\;ij}+u_{j}$$

where $X=\left(X_{1},X_{2},\ldots ,X_{k}\right)$ is the covariate-set and $u_{j}$ is the second-level residual for the macro-area *j*, which is defined as a random variable with zero mean and constant variance. The variables to be included in multivariable models were those statistically significant at the univariable analysis (results only in tables). Data were analyzed using STATA software (IC version 15.0, StataCorp LLC, College Station, TX, USA). A *p*-value of less than 0.05 was considered statistically significant.

## 3. Results

The analyzed sample was mainly composed of women (59%) aged 25–64 years (55%). The majority were married (59%), with high school qualification (33%) followed by middle school qualification (29%). In this sample, the monthly family income was relatively uniformly distributed across quintiles, while the most declared employment status was an employee (35.5%). Fifty-one percent of the interviewed individuals did not have any chronic disease; 54% claimed to have good or very good perceived health, and 84% did not report physical limitations. The majority of the subjects in our sample were from South-Islands (31.9%), followed by North–West (24.5%), North–East (23%), and Centre (20.6%).

Moreover, 52% of the subjects had normal weight and 81% did not smoke currently. Regarding the variables that identify barriers to access, 30% of the subjects reported a delay in performing any health services caused by a long waiting list, and 10% had delayed access to treatment due to distance or unavailability of means of transport. Regarding the costs, 12% declared having difficulties to pay for exams or medical treatment and only 8% for prescribed drugs (Table 1).

Of the 2823 respondents that faced a long waiting list barrier, the majority lived in South-Islands (37.9%), followed by the Centre (24.2%), North–West and North–East (20.1 and 17.8%, respectively). The same ranking was found for all of the other barriers. Of the 902 people facing difficulties due to distance, 46.8% lived in South-Islands, 26.7% in the Centre, 15.6% in North–West, and 10.9% in North–East. Of the 1143 respondents facing an exam cost barrier, the majority were found in South-Islands (46.5%), followed by the Centre (23.5%), North–West and North–East (16.3 and 13.7%, respectively). Finally, of the 725 respondents facing a drugs cost barrier, the majority lived in South-Islands (47.5%), followed by the Centre (22.9%), North–West and North–East (17.7 and 12.0%, respectively).

**Figure 1:**
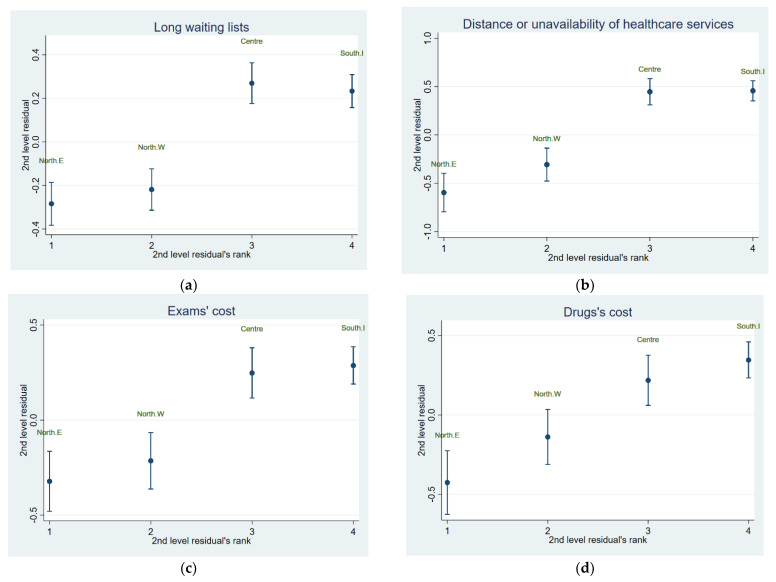
Second level residuals of the variance components of logistic regression models for the delayed utilization due to barriers of (**a**) long waiting list, (**b**) distance or unavailability of means of transport, (**c**) expensive exams, (**d**) expensive prescribed drugs. Legend: North.W = North–West Italy, North.E = North–East Italy, South.I = South-Islands.

The multivariable mixed model for delayed utilization due to a long waiting list showed only income as statistically significant among the socio-economic characteristics. In detail, the increase in income was associated with a reduced risk of delayed utilization due to a long waiting list (adjOR = 0.75, 95% CI (0.65; 0.87) for the 2nd quintile, adjOR = 0.76, 95% CI (0.67; 0.90) for the 3rd quintile, adjOR = 0.77, 95% CI (0.66; 0.90) for the 4th quintile, and adjOR = 0.64, 95% CI (0.55; 0.75) for the 5th quintile, respectively). Among the health status covariates, chronic diseases and physical limitation were negatively associated (adjOR = 1.23, 95% CI (1.10; 1.37) and adjOR = 1.22, 95% CI (1.07; 1.40), respectively), while good self-rated health was positively related with the delay due to a long waiting list (adjOR = 0.83, 95% CI (0.75; 0.93)). Finally, the probability of delayed utilization due to a long waiting list was higher for specialist visits (adjOR = 1.20, 95% CI (1.07; 1.35)). A significant variability, equal to 7%, was found among Italian macro-areas, as shown in Table 2 and Figure 1a.

With regards to distance or unavailability of a mean of transport, the mixed model showed that the probability of delayed utilization decreased by the increasing income (adjOR = 0.71, 95% CI (0.57; 0.87) for the 2nd quintile, adjOR = 0.65, 95% CI (0.52; 0.80) for the 3rd quintile, adjOR = 0.67, 95% CI (0.53; 0.84) for the 4th quintile, and adjOR = 0.47, 95% CI (0.36; 0.60) for the 5th quintile, respectively). In addition, it was lower for subjects with physical limitations (adjOR = 0.81, 95% CI (0.68; 0.97), while it was higher for subjects with good or very good self-perceived health (adjOR = 1.78, 95% CI (1.47; 2.15)). The macro-regions variance of delayed utilization due to difficulties related to distance amounted to 22%, as shown in Table 3 and Figure 1b.

With regards to not performing exams since they are very expensive, the model revealed that males were less exposed to this barrier than females (adjOR = 0.79, 95% CI (0.68; 0.92)). The risk of facing a barrier related to exams’ cost was significant for accessibility to dental visits (adjOR = 0.84, 95% CI (0.73; 0.96)). The higher the income, the lower the probability to be able to afford the cost of exams (adjOR = 0.62, 95% CI (0.52; 0.74), for the 2nd quintile (adjOR = 0.39, 95% CI (0.32; 0.48), for the 3rd quintile (adjOR = 0.33, 95% CI (0.26; 0.41) for the 4th quintile, and adjOR = 0.36, 95% CI (0.28; 0.45) for the 5th quintile, respectively). People with physical limitations were at a major risk of not performing the exams for economic reasons (adjOR = 1.44, 95% CI (1.20; 1.74), while people with good and very good self-perceived health are at a minor risk (adjOR = 0.62, 95% CI (0.52; 0.73)), as shown in Table 4 and Figure 1c.

The last model concerned the impossibility of purchasing the prescribed drugs due to the expensive cost. Similarly, the probability of facing this barrier was lower by the increasing income (adjOR = 0.74, 95% CI (0.60; 0.92) for the 2nd quintile, adjOR = 0.52, 95% CI (0.40; 0.66) for the 3rd quintile, adjOR = 0.51, 95% CI (0.40; 0.67) for the 4th quintile, and adjOR = 0.54, 95% CI (0.41; 0.71) for the 5th quintile, respectively) and for subjects with a good or very good self-perceived health (adjOR = 0.63, 95% CI (0.52; 0.77)). Conversely, it was higher for subjects with physical limitations (adjOR = 1.27, 95% CI (1.02; 1.57)). The significant variability among macro-regions variance amounted to 10%, as shown in Table 5 and Figure 1d.

## 4. Discussion

With the ageing population, equitable access to medical care has proven to be paramount for the effective and efficient management of all chronic diseases [16] and the recent pandemic [17].

Of the four barriers considered in this study, only two were significantly associated with healthcare utilization. Specifically, they are long waiting lists for delayed specialist service accessibility and very expensive exams for dental visit accessibility. Another important result was the evidence of an increasing north–south gradient for all of the considered barriers.

In Italy, the so-called “outpatient waiting time”, which is the time between the GP referral and a specialist visit for successive instrumental and diagnostic investigations, is high and highly variable across regions [18]. Healthcare providers have proposed several solutions in order for waiting lists to be more acceptable and sustainable, such as process re-engineering, the provision of a centralized reservation system, and the implementation of waiting-time prioritization tools [19].

Our results of the cost barriers to dental care are consistent with other studies inside [20] and outside of Europe [21,22]. In Italy, the essential levels of care cover dental visits and procedures only if they are addressed to the pediatric age or to specific categories of particularly vulnerable persons, such as oncologic patients or if the care is necessitated by an acute event, such as inflammation or bleeding. Dental care is financed through private insurance and/or out-of-pocket expenditures in all of the other cases.

Waiting lists, distance, cost of examinations, and cost of drugs showed a similar pattern with respect to income and health indicators. In fact, the more income decreases, the higher the probability of experiencing a barrier to healthcare access. This association is evident for the elderly as it has been shown that the worse socio-economic status results in reduced access to social support networks and increased perceived social isolation, which makes it more difficult to access healthcare [23,24,25].

Furthermore, people with good self-perceived health are less likely to report one healthcare barrier among waiting lists, distance, instrumental diagnostic exams or drug prescriptions. At the same time, those affected by chronic diseases have a higher likelihood of experiencing a healthcare barrier, as mentioned above [26]. Indeed, in a Greek study, it was estimated that 63.5 and 58.5% of chronic patients reported economic and waiting list barriers, respectively [27]. Specifically, low income, low-educated, and unemployed people are more likely to face economic barriers to access along with people affected by chronic diseases.

Regarding waiting lists, the OECD report supports our results that this barrier is more common among people with low income in more than half of the countries [28]. Moreover, we did not find an association between waiting lists and education, in accordance with the other literature [29].

Waiting times could be detrimental to health in general and to patients’ quality of life. Delays in accessing services due to a long waiting list can impact the psychological health of patients that can report negative feelings, such as abandonment, frustration, anxiety, and distress. In addition, it can affect the health treatment outcomes or be detrimental for health conditions. Long waiting times could worsen the disease status, the physical conditions or the activity limitations. They can also impact mental health and worsen some statuses, such as depression or substance addiction [30]. Moreover, they can be more dangerous for patients waiting for time-sensitive procedures (e.g., cardiac or cancer surgery) [31,32,33].

Long waiting times are not only associated with worse health, but are responsible for an increase in health inequalities. In fact, high-income patients can overcome the barriers to access, resorting to the private sector as they can afford to pay for healthcare outside of the public healthcare frame [27].

Our study showed the higher probability of facing a barrier due to exams’ costs for females and people with a low income, in agreement with the other literature [34]. Regarding gender gaps, it can be viewed as the result of gender differences in the Italian labor market [35]. It is well documented that on average, women earn 14.1% less per hour than men for the same position and seniority, show higher unemployment rates (20%), and have high-skilled and highly paid employment that is less than men [36,37]. Lower economic availability lowers the ability to access expensive diagnostic and instrumental examinations. The socio-economic differences between sexes to access exams is wider as we move along the Italian north–south trajectory: In Southern-Italy, women work less, have lower levels of education, and have fewer and lesser quality childcare and parental services. A similar pattern was found in another work of the research group, where the increasing north–south gradient of health was ascribed to the deteriorating socio-economic and cultural context, as we move downward along Italy [38].

Interestingly, in our paper, the distance of a service was not significantly associated with hospitalizations, GP, specialists, and dental visits. This result can be explained by specific characteristics of the healthcare system in Italy. In fact, the Italian legislation establishes the right of citizens to be treated in any healthcare facility, even outside of the region of residence. Following the “health federalism”, many regions without facilities that are suitable for treating certain diseases have introduced regulations to allow for patients to undergo a treatment in other Italian regions and receive refunds, including expenses for accommodation, travel expenses, and expenses for the purchase of drugs. Agreement No. 82 of the State-Region Conference of 10 July 2014, concerning the new Health Pact for the years 2014–2016, lastly updated on 31 March 2020, establishes the rules for the compensation of health mobility.

Our study found that income exacerbates the role of the costs of prescribed drugs as a barrier to healthcare access. This circumstance is quite common in European countries, where some people may face problems accessing prescribed drugs or diagnostics exams due to financial issues [39]. The costs of prescribed drugs represent a severe barrier as they influence physicians’ therapeutic recommendations and patients’ compliance, especially in oncology [40].

Regarding all of the barriers to healthcare access, physical limitation was an important factor. Some recent literature showed that the uneven geographical distribution of medical facilities, the difficulty of moving to hospitals, and restrictions of communication within the health care system increase the probability of a barrier for people with physical disabilities compared to the counterpart without a disability due to the socio-economic status or transportation problem [41,42].

The final important result of our study was the significant heterogeneity among the Italian macro-areas regarding individuals’ access to healthcare. This extent of variation can be due to the organizational differences among regional healthcare systems, regarding the territorial distribution of services and the quality of structural equipment between North and South [39]. These differences have intensified with the outbreak of the 2019 pandemic. While high income regions have been able to supply higher quality healthcare to a wide range of patients, regardless of their socio-economic profile, in low-income regions, the health offer has often been inadequate for lack of intensive care beds and protective equipment for medical and nursing staff [43]. Through an understanding of the socio-economic determinants of healthcare utilization and inequality in the barriers to access, the health policy can pursue equity in the response to all of the chronic diseases and to the current health emergency caused by COVID-19.

To draw the right conclusions from the study, some aspects need to be considered First, the study sample includes only people in need of healthcare and, consequently, the percentage of very young respondents or those with physical limitations is quite low. Therefore, the study sample does not reflect the structure of the Italian population in 2015. Second, the present analysis does not consider the question of individuals facing multiple barriers at the same time. In addition, the fact that there are multiplicative effects on healthcare inequality cannot be excluded, but this question needs to be further investigated. Third, it would be interesting to investigate the role of distrust in public health care services as an additional access barrier as well as the interlinked financial, cognitive, and structural barriers in internal migration to health care access. Unfortunately, the EHIS data did not consider this question.

Finally, one important question to be further investigated is the role of COVID-19 as a barrier to healthcare utilization. A recent study in Lombardy [44] showed up to 73% (95% CI: 63–80%) of healthcare reductions during the lockdown for timeliness of breast cancer surgery, and up to 20% (95% CI: 16–23%) for appropriated gynaecologic visit during pregnancy. Estimating the change in the use of primary, outpatient specialists and dental services, in addition to hospitalizations is the objective of our next research.

## 5. Conclusions

This study demonstrated the existence of socio-economic inequality in the utilization of specialist and dental visits, while visits to the GP and hospitalizations did not suffer any barriers. Long waiting lists were found to be the most important barriers for specialist visits, while financial barriers were found to be most important for dental visits. Finally, there was significant heterogeneity throughout Italy, and barriers to healthcare access showed a north–south gradient distribution. Furthermore, interventions aimed at reducing structural inequalities among the Italian regions are desirable in order to achieve equity in access to care, particularly to cope with new health emergencies.

## Figures and Tables

**Table 1:** Demographic, socio-economic characteristics, health status, and barriers to healthcare access of 9629 Italian individuals, aged 15 and over.

Variables		*n* (%)
**Demographic and socio-economic characteristics**		
Gender	Male	3987 (41%)
	Female	5642 (59%)
Age	15–24	563 (6%)
	25–64	5289 (55%)
	65+	3777 (39%)
Marital status	Unmarried	3903 (41%)
	Married	5726 (59%)
Educational level	No qualification	2395 (25%)
	Middle school	2810 (29%)
	High school	3156 (33%)
	Graduation	1268 (13%)
Income	1st quintile	1605 (17%)
	2nd quintile	1920 (20%)
	3rd quintile	1992 (21%)
	4th quintile	2050 (21%)
	5th quintile	2062 (21%)
Labor status	Unemployed	662 (6.9%)
	Employed	3418 (35.5%)
	Retired	3004 (31.2%)
	Other	2545 (26.4%)
Macro-regions	North–West	2358 (24.5%)
	North–East	2216 (23.0%)
	Centre	1986 (20.6%)
	South-Islands	3069 (31.9%)
**Health status**		
Chronic disease	None	4812 (51%)
	At least one	4657 (49%)
Self-rated health	Less than good	4365 (46%)
	Good and very good	5108 (54%)
Physical limitation	None	8061 (84%)
	At least one	1568 (16%)
Weight status	Underweight/Normal	4948 (52%)
	Overweight	3318 (34%)
	Obese	1205 (13%)
Smoking history	Not a smoker	7648 (81%)
	Smoker	1789 (19%)
**Barriers to healthcare utilization**		
Long waiting list	No	6545 (70%)
	Yes	2823 (30%)
Difficulties due to distance or transport	No	8471 (90%)
	Yes	902 (10%)
Do not perform exams or medical treatments since they are very expensive	No	8237 (88%)
	Yes	1143 (12%)
Do not take drugs since they are very expensive	No	8655 (92%)
	Yes	725 (8%)

**Table 2:** Delayed utilization due to a long waiting list related to the socio-economic and health characteristics of respondents: Univariable and multivariable analyses.

Variables		OR (95% CI)	adjOR (95% CI)
**Demographic and socio-economic characteristics**			
Gender	Male vs. female	0.90 (0.82; 0.98)	0.95 (0.86; 1.06)
Age	25–64 vs. 15–24	1.44 (1.17; 1.77)	1.27 (1.01; 1.60)
	65+ vs. 15–24	1.38 (1.12; 1.71)	1.07 (0.83; 1.37)
Marital status	Married vs. unmarried	0.97 (0.89; 1.06)	
Educational level	Middle school vs. No qualification	0.95 (0.84; 1.07)	
	High school vs. No qualification	0.91 (0.81; 1.02)	
	Graduation vs. No qualification	0.90 (0.77; 1.05)	
Income	2nd vs. 1st quintile	0.74 (0.64; 0.85)	0.75 (0.65; 0.87)
	3rd vs. 1st quintile	0.70 (0.61; 0.81)	0.76 (0.67; 0.90)
	4th vs. 1st quintile	0.66 (0.57; 0.76)	0.77 (0.66; 0.90)
	5th vs. 1st quintile	0.54 (0.47; 0.62)	0.64 (0.55; 0.75)
Labor status	Employed vs. unemployed	0.72 (0.60; 0.86)	0.90 (0.74; 1.09)
	Retired vs. unemployed	0.74 (0.61; 0.88)	0.87 (0.75; 1.09)
	Other vs. unemployed	0.86 (0.71; 1.03)	0.87 (0.71; 1.07)
Macro-regions	North–East vs. North–West	0.92 (0.80; 1.06)	
	Centre vs. North–West	1.65 (1.44; 1.88)	
	South-Islands vs. North–West	1.73 (1.53; 1.95)	
**Health status**			
Chronic disease	At least one vs. None	1.40 (1.28; 1.53)	1.23 (1.10; 1.37)
Self-rated health	Good and very good vs. Less than good	0.68 (0.62; 0.74)	0.83 (0.75; 0.93)
Physical limitation	At least one vs. None	1.44 (1.28; 1.61)	1.22 (1.07; 1.40)
Weight status	Overweight vs. Underweight/Normal	1.09 (0.99; 1.21)	1.03 (0.92; 1.14)
	Obese vs. Underweight/Normal	1.38 (1.20; 1.57)	1.19(1.03; 1.37)
Smoking history	Smoker vs. not a smoker	1.09 (0.97; 1.22)	
**Healthcare Utilization**			
Hospitalization	Yes vs. no	1.15 (1.02; 1.30)	0.97 (0.85; 1.10)
GP visits	Yes vs. no	1.37 (1.16; 1.62)	1.15 (0.96; 1.36)
Specialist visits	Yes vs. no	1.25 (1.12; 1.39)	1.20 (1.07; 1.35)
Dental visits	Yes vs. no	0.99 (0.90; 1.07)	
**Macro-regions variance**			0.07 (0.02; 0.28)

**Table 3:** Delayed utilization due to distance difficulties related to socio-economic and health characteristics of respondents: Univariable and multivariable analyses.

Variables		OR (95% CI)	adjOR (95% CI)
**Demographic and socio-economic characteristics**			
Gender	Male vs. female	0.90 (0.78; 1.04)	
Age	25–64 vs. 15–24	1.64 (1.12; 2.40)	1.52 (1.03; 2.28)
	65+ vs. 15–24	2.06 (1.40; 3.02)	1.46 (0.96; 2.24)
Marital status	Married vs. unmarried	0.89 (0.75; 1.02)	
Educational level	Middle school vs. No qualification	0.76 (0.64; 0.91)	0.98 (0.80; 1.21)
	High school vs. No qualification	0.51 (0.43; 0.62)	0.78 (0.62; 0.97)
	Graduation vs. No qualification	0.58 (0.46; 0.74)	0.96 (0.72; 1.28)
Income	2nd vs. 1st quintile	0.71 (0.58; 0.86)	0.71 (0.57, 0.87)
	3rd vs. 1st quintile	0.57 (0.46; 0.70)	0.65 (0.52; 0.80)
	4th vs. 1st quintile	0.51 (0.42; 0.63)	0.67 (0.53; 0.84)
	5th vs. 1st quintile	0.34 (0.27; 0.44)	0.47 (0.36; 0.60)
Labor status	Employed vs. unemployed	0.71 (0.53; 1.94)	
	Retired vs. unemployed	0.87 (0.65; 1.16)	
	Other vs. unemployed	1.21 (0.91; 1.60)	
Macro-regions	North–East vs. North–West	0.72 (0.56; 0.95)	
	Centre vs. North–West	2.17 (1.74; 2.70)	
	South-Islands vs. North–West	2.55 (2.08; 3.12)	
**Health status**			
Chronic disease	At least one vs. None	1.34 (1.17; 1.54)	0.96 (0.81; 1.14)
Self-rated health	Good and very good vs. Less than good	0.57 (0.50; 0.66)	0.81 (0.68; 0.97)
Physical limitation	At least one vs. None	2.19 (1.87; 2.57)	1.78 (1.47; 2.15)
Weight status	Overweight vs. Underweight/Normal	1.25 (1.08; 1.46)	1.07 (0.91; 1.25)
	Obese vs. Underweight/Normal	1.47 (1.20; 1.80)	1.13 (0.91; 1.40)
Smoking history	Smoker vs. not a smoker	1.05 (0.88; 1.25)	
**Healthcare Utilization**			
Hospitalization	Yes vs. no	1.30 (1.09; 1.56)	
GP visits	Yes vs. no	1.13 (0.88; 1.46)	
Specialist visits	Yes vs. no	0.96 (0.82; 1.13)	
Dental visits	Yes vs. no	0.75 (0.65; 0.86)	0.96 (0.83; 1.12)
**Macro-regions variance**			0.22 (0.05; 0.93)

**Table 4:** Not performing exams since they are very expensive related to socio-economic and health characteristics of respondents: Univariable and multivariable analyses.

Variables		OR (95% CI)	AdjOR (95% CI)
**Demographic and socio-economic characteristics**			
Gender	Male vs. female	0.74 (0.65; 0.84)	0.79 (0.68; 0.92)
Age	25–64 vs. 15–24	1.37 (1.03; 1.84)	1.26 (0.90; 1.75)
	65+ vs. 15–24	1.01 (0.75; 1.36)	0.95 (0.65; 1.38)
Marital status	Married vs. unmarried	0.94 (0.83; 1.06)	
Educational level	Middle school vs. No qualification	0.99 (0.84; 1.16)	0.96 (0.79; 1.17)
	High school vs. No qualification	0.79 (0.67; 0.93)	0.94 (0.76; 1.17)
	Graduation vs. No qualification	0.47 (0.37; 0.60)	0.63 (0.46; 0.85)
Income	2nd vs. 1st quintile	0.57 (0.48; 0.67)	0.62 (0.52; 0.74)
	3rd vs. 1st quintile	0.32 (0.26; 0.39)	0.39 (0.32; 0.48)
	4th vs. 1st quintile	0.24 (0.19; 0.29)	0.33 (0.26; 0.41)
	5th vs. 1st quintile	0.23 (0.19; 0.29)	0.36 (0.28; 0.45)
Labor status	Employed vs. unemployed	0.51 (0.41; 0.63)	0.95 (0.74; 1.22)
	Retired vs. unemployed	0.34 (0.27; 0.43)	0.52 (0.38; 0.70)
	Other vs. unemployed	0.76 (0.61; 0.95)	0.79 (0.61; 1.03)
Macro-regions	North–East vs. North–West	0.88 (0.71; 1.10)	
	Centre vs. North–West	1.82 (1.49; 2.22)	
	South-Islands vs. North–West	2.46 (2.06; 2.94)	
**Health status**			
Chronic disease	At least one vs. None	1.34 (1.18; 1.52)	1.03 (0.88; 1.21)
Self-rated health	Good and very good vs. Less than good	0.54 (0.48; 0.61)	0.62 (0.52; 0.73)
Physical limitation	At least one vs. None	1.63 (1.41; 1.90)	1.44 (1.20; 1.74)
Weight status	Overweight vs. Underweight/Normal	1.20 (1.05; 1.37)	1.17 (1.00; 1.35)
	Obese vs. Underweight/Normal	1.42 (1.18; 1.70)	1.13 (0.92; 1.38)
Smoking history	Smoker vs. not a smoker	1.23 (1.06; 1.43)	1.14 (0.97; 1.35)
**Healthcare Utilization**			
Hospitalization	Yes vs. no	1.32 (1.12; 1.55)	1.08 (0.90; 1.29)
GP visits	Yes vs. no	1.39 (1.09; 1.77)	1.30 (1.00; 1.70)
Specialist visits	Yes vs. no	1.01 (0.87; 1.18)	
Dental visits	Yes vs. no	0.70 (0.62; 0.80)	0.84 (0.73; 0.96)
**Macro-regions variance**			0.08 (0.02; 0.35)

**Table 5:** Not purchasing drugs since they are very expensive related to socio-economic and health characteristics of respondents: Univariable and multivariable analyses.

Variables		OR (95% CI)	AdjOR (95% CI)
**Demographic and socio-economic characteristics**			
Gender	Male vs. female	0.84 (0.71; 0.98)	0.96 (0.80; 1.15)
Age	25–64 vs. 15–24	1.15 (0.81; 1.63)	
	65+ vs. 15–24	1.10 (0.77; 1.56)	
Marital status	Married vs. unmarried	0.90 (0.77; 1.05)	
Educational level	Middle school vs. No qualification	0.87 (0.72; 1.06)	0.95 (0.76; 1.18)
	High school vs. No qualification	0.65 (0.54; 0.80)	0.85 (0.67; 1.08)
	Graduation vs. No qualification	0.52 (0.39; 0.69)	0.74 (0.53; 1.04)
Income	2nd vs. 1st quintile	069 (0.56; 0.85)	0.74 (0.60; 0.92)
	3rd vs. 1st quintile	0.41 (0.32; 0.52)	0.52 (0.40; 0.66)
	4th vs. 1st quintile	0.36 (0.28; 0.46)	0.51 (0.40; 0.67)
	5th vs. 1st quintile	0.34 (0.26; 0.43)	0.54 (0.41; 0.71)
Labor status	Employed vs. unemployed	0.49 (0.37; 0.64)	0.77 (0.57; 1.04)
	Retired vs. unemployed	0.45 (0.34; 0.60)	0.52 (0.38; 0.71)
	Other vs. unemployed	0.86 (0.66; 1.13)	0.83 (0.62; 1.12)
Macro-regions	North–East vs. North–West	0.71 (0.53; 0.93)	
	Centre vs. North–West	1.58 (1.24; 2.01)	
	South-Islands vs. North–West	2.21 (1.79; 2.73)	
**Health status**			
Chronic disease	At least one vs. None	1.20 (1.03; 1.40)	0.89 (0.74; 1.07)
Self-rated health	Good and very good vs. Less than good	0.57 (0.48; 0.66)	0.63 (0.52; 0.77)
Physical limitation	At least one vs. None	1.56 (1.30; 1.88)	1.27 (1.02; 1.57)
Weight status	Overweight vs. Underweight/Normal	1.18 (1.00; 1.39)	1.10 (0.92; 1.31)
	Obese vs. Underweight/Normal	1.45 (1.16; 1.81)	1.17 (0.93; 1.48)
Smoking history	Smoker vs. not a smoker	1.19 (0.99; 1.43)	
**Healthcare Utilization**			
Hospitalization	Yes vs. no	1.16 (0.94; 1.42)	
GP visits	Yes vs. no	1.14 (0.86; 1.50)	
Specialist visits	Yes vs. no	0.96 (0.80; 1.15)	
Dental visits	Yes vs. no	0.71 (0.61; 0.83)	0.87 (0.74; 1.01)
**Macro-regions variance**			0.10 (0.02; 0.45)

## Data Availability

Data are available upon request from Eurostat.

## References

[1] Ferré F., de Belvis A.G., Valerio L., Longhi S., Lazzari A., Fattore G., Ricciardi W., Maresso A. (2014). Italy: Health system review. Health Syst. Transit..

[2] Matranga D., Bono F., Casuccio A., Firenze A., Marsala L., Giaimo R., Sapienza F., Vitale F. (2014). Evaluating the effect of organization and context on technical efficiency: A second-stage DEA analysis of Italian hospitals. Epidemiol. Biostat. Public Health.

[3] Matranga D., Sapienza F. (2015). Congestion analysis to evaluate the efficiency and appropriateness of hospitals in Sicily. Health Policy.

[4] Allin S., Masseria C., Sorenson C., Papanicola I., Mossialos E. (2007). Measuring Inequalities in Access to Health Care: A Review of the Indices. Lond. Sch. Econ. Polit. Sci..

[5] Allin S., Masseria C., Mossialos E. (2009). Measuring socioeconomic differences in use of health care services by wealth versus by income. Am. J. Public Health.

[6] Lueckmann S.L., Hoebel J., Roick J., Markert J., Spallek J., von dem Knesebeck O., Richter M. (2021). Socioeconomic inequalities in primary-care and specialist physician visits: A systematic review. Int. J. Equity Health.

[7] van Doorslaer E., Koolman X., Jones A.M. (2004). Explaining income-related inequalities in doctor utilisation in Europe. Health Econ..

[8] Lostao L., Geyer S., Albaladejo R., Moreno-Lostao A., Santos J.M., Regidor E. (2017). Socioeconomic position and health services use in Germany and Spain during the Great Recession. PLoS ONE.

[9] Hoebel J., Rattay P., Prütz F., Rommel A., Lampert T. (2016). Socioeconomic Status and Use of Outpatient Medical Care: The Case of Germany. PLoS ONE.

[10] Masseria C., Giannoni M. (2010). Equity in access to health care in Italy: A disease-based approach. Eur. J. Public Health.

[11] Devakumar D., Bhopal S.S., Shannon G. (2020). COVID-19: The great unequaliser. J. R. Soc. Med..

[12] Costa G., Marinacci C., Caiazzo A., Spada T. (2003). Individual and contextual determinants of inequalities in health: The Italian case. Int. J. Health Serv..

[13] Shen J., Listl S. (2018). Investigating social inequalities in older adults’ dentition and the role of dental service use in 14 European countries. Eur. J. Health Econ..

[14] Commission E.E. European Health Interview Survey (EHIS Wave 2) Methodological Manual. Publications Office of the European Union. https://ec.europa.eu/eurostat/web/products-manuals-and-guidelines/-/KS-RA-13-018.

[15] (2018). Italian National Institute of Statistics. https://www.istat.it/it/archivio/225274.

[16] Jo O., Kruger E., Tennant M. (2021). GIS mapping of healthcare practices: Do older adults have equitable access to dental and medical care in the UK?. Br. Dent. J..

[17] Matranga D., Maniscalco L., Enea M., De Luca D., Brancato D., La Spada E., Scorsone A., Di Carlo P. Longitudinal investigation of severe acute respiratory syndrome coronavirus 2 (SARS-CoV-2) infection in older patients in the province of Palermo (Southern Italy) during the early wave of the pandemic. Arch. Med. Sci..

[18] Mariotti G., Siciliani L., Rebba V., Fellini R., Gentilini M., Benea G., Bertoli P., Bistolfi L., Brugaletta S., Camboa P. (2014). Waiting time prioritisation for specialist services in Italy: The homogeneous waiting time groups approach. Health Policy.

[19] Norheim O.F. (2008). Clinical priority setting. BMJ.

[20] Holm-Pedersen P., Vigild M., Nitschke I., Berkey D.B. (2005). Dental care for aging populations in Denmark, Sweden, Norway, United kingdom, and Germany. J. Dent. Educ..

[21] Thompson B. (2012). Cost Barriers to Dental Care in Canada.

[22] Thompson B., Cooney P., Lawrence H., Ravaghi V., Quiñonez C. (2014). The potential oral health impact of cost barriers to dental care: Findings from a Canadian population-based study. BMC Oral Health.

[23] Berkman L.F. (1995). The role of social relations in health promotion. Psychosom. Med..

[24] Miceli S., Maniscalco L., Matranga D. (2019). Social networks and social activities promote cognitive functioning in both concurrent and prospective time: Evidence from the SHARE survey. Eur. J. Ageing.

[25] Misuraca R., Miceli S., Teuscher U. (2017). Three effective ways to nurture our brain: Physical activity, healthy nutrition, and music. Rev. Eur. Psychol..

[26] Cavalieri M. (2013). Geographical variation of unmet medical needs in Italy: A multivariate logistic regression analysis. Int. J. Health Geogr..

[27] Kyriopoulos I.-I., Zavras D., Skroumpelos A., Mylona K., Athanasakis K., Kyriopoulos J. (2014). Barriers in access to healthcare services for chronic patients in times of austerity: An empirical approach in Greece. Int. J. Equity Health.

[28] OECD (2019). OECD Health Policy Studies Health for Everyone?. Social Inequalities in Health and Health Systems.

[29] Moran V., Suhrcke M., Ruiz-Castell M., Barré J., Huiart L. (2021). Investigating unmet need for healthcare using the European Health Interview Survey: A cross-sectional survey study of Luxembourg. BMJ Open.

[30] van Beljouw I.M.J., Verhaak P.F.M., Cuijpers P., van Marwijk H.W.J., Penninx B.W.J.H. (2010). The course of untreated anxiety and depression, and determinants of poor one-year outcome: A one-year cohort study. BMC Psychiatry..

[31] Gagliardi A.R., Yip C.Y.Y., Irish J., Wright F.C., Rubin B., Ross H., Green R., Abbey S., McAndrews M.P., Stewart D.E. (2021). The psychological burden of waiting for procedures and patient-centred strategies that could support the mental health of wait-listed patients and caregivers during the COVID-19 pandemic: A scoping review. Health Expect..

[32] Flaherty L.B., Wood T., Cheng A., Khan A.R. (2017). Pre-existing psychological depression confers increased risk of adverse cardiovascular outcomes following cardiac surgery: A systematic review and meta-analysis. J. Thorac. Cardiovasc. Surg..

[33] Celano C.M., Villegas A.C., Albanese A.M., Gaggin H.K., Huffman J.C. (2018). Depression and anxiety in heart failure: A review. Harv. Rev. Psychiatry.

[34] Fiorillo D. (2020). Reasons for unmet needs for health care: The role of social capital and social support in some Western EU countries. Int. J. Health Econ. Manag..

[35] Kühn S., Horne R., Yoon S. (2017). World Employment and Social Outlook: Trends for Women 2017.

[36] Landmesser J.M., Orłowski A.J., Rusek M.A. (2020). Gender Pay Gap across the Income Distribution: Analysis for the EU. Acta Phys. Pol. A.

[37] Publishing O. (2017). The Pursuit of Gender Equality—An Uphill Battle.

[38] Matranga D., Tabacchi G., Cangialosi D. (2018). Sedentariness and weight status related to SES and family characteristics in Italian adults: Exploring geographic variability through multilevel models. Scand. J. Public Health.

[39] Baeten R., Spasova S., Vanhercke B., Coster S. (2018). Inequalities in Access to Healthcare—A Study of National Policies.

[40] Neumann P.J., Palmer J.A., Nadler E., Fang C., Ubel P. (2010). Cancer therapy costs influence treatment: A national survey of oncologists. Health Aff..

[41] Hwang B., Chun S.-M., Park J.-H., Shin H.-I. (2011). Unmet healthcare needs in people with disabilities: Comparison with the general population in Korea. Ann. Rehabil. Med..

[42] McColl M.A., Jarzynowska A., Shortt S.E.D. (2010). Unmet health care needs of people with disabilities: Population level evidence. Disabil. Soc..

[43] Shadmi E., Chen Y., Dourado I., Faran-Perach I., Furler J., Hangoma P., Hanvoravongchai P., Obando C., Petrosyan V., Rao K.D. (2020). Health equity and COVID-19: Global perspectives. Int. J. Equity Health.

[44] Corrao G., Cantarutti A., Compagnoni M.M., Franchi M., Rea F. (2022). Change in healthcare during COVID-19 pandemic was assessed through observational designs. J. Clin. Epidemiol..

